# Glaucocalyxin A exerts anticancer effect on osteosarcoma by inhibiting GLI1 nuclear translocation via regulating PI3K/Akt pathway

**DOI:** 10.1038/s41419-018-0684-9

**Published:** 2018-06-13

**Authors:** Jianwei Zhu, Yang Sun, Ying Lu, Xiubo Jiang, Bo Ma, Lisha Yu, Jie Zhang, Xiaochen Dong, Qi Zhang

**Affiliations:** 1 School of Pharmaceutical Sciences, Nanjing Tech University (NanjingTech), 30 South Puzhu Road, Nanjing, 211816 China; 2 Key Laboratory of Flexible Electronics (KLOFE) & Institute of Advanced Materials (IAM), Jiangsu National Synergetic Innovation Center for Advanced Materials (SICAM), Nanjing Tech University (NanjingTech), 30 South Puzhu Road, Nanjing, 211816 China

## Abstract

Osteosarcoma, the most common malignant bone tumor with recurring disease or lung metastases, has become one of the leading causes of death in humans. In the current study, we made an investigation on the anticancer effect of glaucocalyxin A, a bioactive ent-kauranoid diterpenoid isolated from *Rabdosia japonica* var., and unraveled the underlying mechanisms. Here, we found that Glaucocalyxin A inhibited the cell viability of numerous osteosarcoma cells. Our results showed that Glaucocalyxin A exerted the pro-apoptotic effect on human osteosarcoma cells, MG-63 and HOS cells. Glaucocalyxin A induced apoptosis by mitochondrial apoptotic pathway through several steps including increasing the Bax/Bcl-2 ratio, triggering the intracellular reactive oxygen species (ROS) generation, reducing mitochondrial membrane potential (MMP), and inducing cleavage of caspase-9 and caspase-3. We demonstrated that Glaucocalyxin A induced apoptosis via inhibiting Five-zinc finger Glis 1 (GLI1) activation by overexpression and knockdown of GLI1 in vitro. We also found that Glaucocalyxin A inhibited GLI1 activation via regulating phosphatidylinositol 3 kinase/protein kinase B (PI3K/Akt) signaling pathway. We further confirmed our findings by using PI3K activator and inhibitor to verify the inhibitory effect of Glaucocalyxin A on PI3K/Akt/GLI1 pathway. Moreover, our in vivo study revealed that glaucocalyxin A possessed a remarkable antitumor effect with no toxicity in the xenograft model inoculated with HOS tumor through the same mechanisms as in vitro. In conclusion, our results suggested that Glaucocalyxin A induced apoptosis in osteosarcoma by inhibiting nuclear translocation of GLI1 via regulating PI3K/Akt signaling pathway. Thus, Glaucocalyxin A might be a potential candidate for human osteosarcoma in the future.

## Introduction

Osteosarcoma, a prevailing primary bone cancer among adolescents and young adults, has become a high risk for death in humans. Although there are lower-grade variants, most of them are high-grade malignancies for lung metastases at a high propensity^1^. Recently, the standard treatment consists of surgical resection and chemotherapy leading to nearly 60% of patients with local extremity disease^2–5^ and 20−30% of patients with primary metastases^2,5^. Preoperative and postoperative chemotherapy, as well as surgical excision are commonly adopted to treat high-grade osteosarcomas; however, a very limited number of drugs are long-time available for the adverse effect and toxicity. Therefore, it is urgent to develop novel effective therapeutic agents for osteosarcoma.

Increasing evidence has reported that phosphoinositide 3-kinase/protein kinase B (PI3K/Akt) pathway contributes to cancer initiation and development, such as tumorigenesis, inhibition of apoptosis, proliferation, and chemoresistance^6^. PI3K/Akt pathway can enhance the tolerance of cells to hypoxia and nutritional deficiencies through the inhibition of apoptosis, so that it is related to the development of breast cancer, lung cancer, melanoma, lymphoma, and other human tumors^7–10^. PI3K could catalytically induce the production of the lipid second messenger phosphatidylinositol-3,4,5-triphosphate (PIP3) at the cell membrane, leading to the recruitment and activation of the downstream targets, such as the serine-threonine protein kinase Akt^11^. Akt phosphorylation plays a crucial role in the anti-apoptotic pathway. Akt can be activated by insulin-like growth factor 1 (IGF1) and prevents PTEN-mediated apoptosis^12,13^. Akt activation also plays an anti-apoptotic role by phosphorylating the downstream target proteins, such as Bcl-2 and caspase-3 and then prevent apoptosis^14^. The downstream proteins of PI3K/Akt pathway mainly regulate apoptosis on the outer membrane of mitochondria and control the initiation of mitochondrial outer membrane permeabilization^15^. Moreover, PI3K/Akt pathway is frequently hyperactivated in osteosarcoma^16^. Inhibiting PI3K/Akt signaling pathway leads to increased apoptotic cells in osteosarcoma via downregulation of the inhibitor of apoptosis protein and activation of caspase-9 and caspase-3 ^17^. Therefore, targeting PI3K/Akt pathway has commanded a great deal of recent attention for the development of anticancer agents.

Hedgehog signaling pathway has an essential impact on the formation of most tissues and organs in mammals, such as cell growth and survival, cell fate determination and organ morphogenesis^18–21^, and it is closely related to the development of human tumors. The intracellular factors involved in Hedgehog signaling transduction include transcription factor Cubitus Interruptus (CI)/five-zinc finger Glis (GLI)^22^. GLI (GLI1 and GLI2), as a crucial transcription factor in the Hedgehog signaling pathway, regulates the transcription of multiple downstream target genes and promote tumor progression. Studies from many laboratories have found the activation of GLI in a variety of human cancer, including basal cell carcinomas, medulloblastomas, leukemia, gastrointestinal, lung, ovarian, breast, and prostate cancers^19,23–25^. It is thus believed that targeted inhibition of GLI may be effective in the treatment and prevention of human cancer. It has been documented that GLI enabled to promote the development of osteosarcoma^26^. The nuclear translocation of GLI can induce the expression of various context-specific genes, for example, encoding the D-type cyclins, c-MYC (also called MYC), BCL2 and SNAIL (also called SNAI1), which respectively regulated cellular differentiation, proliferation, and survival^16,18,26,27^. The GLI1/Bcl-2 pathway is related to anti-apoptosis, with accompanying of the caspase cascade deregulation^28^. Non-canonical GLI1 activation is regulated by PI3K/Akt signaling pathway and inhibiting PI3K/Akt/GLI1 pathway can induce apoptosis and suppress the growth of renal cell carcinoma in vitro and in vivo^29^. Additionally, recent study has reported that PI3K/Akt leads to the activation of GLI1 in human esophageal adenocarcinoma cells OE19 in vitro^30^. Therefore, chemotherapy targeting PI3K/Akt pathway can inhibit GLI activation to prevent cancer.

Glaucocalyxin A, an ent-kauranoid diterpene from *Rabdosia japonica* var., is known to possess numerous biological activities including inhibition of platelet aggregation^31^, immunosuppressive activity, antioxidative and DNA damage protective activity, and cytotoxic activity^32^. It has been documented that Glaucocalyxin A induced apoptosis in human leukemia HL-60 cells and human breast cancer cells^31,33^. Moreover, Glaucocalyxin A suppressed cell proliferation and promoted apoptosis in a dose-dependent manner in human-derived malignant glioma U87MG cells^32^. However, the anticancer effect of Glaucocalyxin A on osteosarcoma has not been reported till now and the potential mechanisms still remain unclear. In the present study we investigated the anticancer effect of Glaucocalyxin A on human osteosarcoma and the underlying mechanisms. We demonstrated that Glaucocalyxin A exerted a dramatic pro-apoptotic effect by inhibiting GLI1 activation via regulating PI3K/Akt signaling pathway in vitro and in vivo. Our findings indicated that Glaucocalyxin A might have an attractive advantage to be a promising and effective candidate for human osteosarcoma in the future.

## Results

### Glaucocalyxin A induced apoptosis in human osteosarcoma cells

**Fig. 1:**
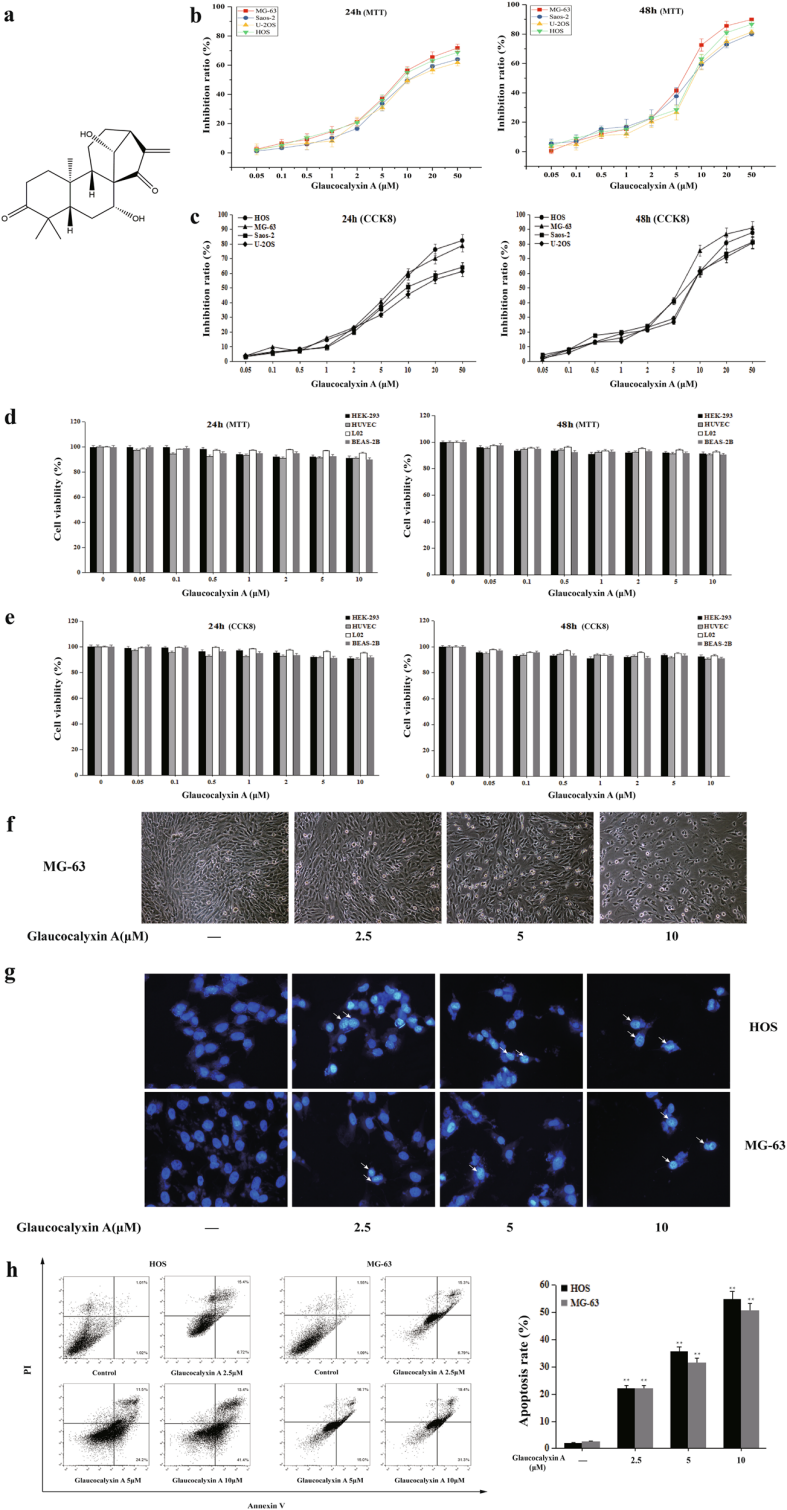
Glaucocalyxin A inhibited the cell viability and induced apoptosis in human osteosarcoma cells. **a** The chemical structure of Glaucocalyxin A. **b**, **c** MTT and CCK8 assays were used to detect the cell viability of human osteosarcoma cells after different concentrations of Glaucocalyxin A for 24 and 48 h respectively. **d**, **e** MTT and CCK8 assays were used to detect the cell viability of human normal cells after different concentrations of Glaucocalyxin A for 24 and 48 h. **f** MG-63 cells were observed under an inverted light microscope after treatment with 2.5, 5 and 10 μM Glaucocalyxin A for 24 h (×200). **g** The morphology of nucleus in HOS and MG-63 cells was observed by DAPI staining (×400). **h** The apoptotic rates of HOS and MG-63 cells induced by Glaucocalyxin A were detected by Annexin V/PI double-staining assay. The results are shown as means ± SD from three independent experiments. ^**^P < 0.01 compared with control group

The chemical structure of Glaucocalyxin A is shown in Fig. 1a. We evaluated the inhibitory effect of Glaucocalyxin A on the cell viability of human osteosarcoma cells including HOS, Saos-2, U-2OS, and MG-63 cells after different concentrations of Glaucocalyxin A for 24 and 48 h. After treatment with Glaucocalyxin A for 24 h, the IC_50_ (the concentration of drug inhibiting 50% of cells) values of HOS, Saos-2, U-2OS, and MG-63 cells were 10.65, 14.14, 15.69, and 9.519 μM, respectively (Fig. 1b). After treatment for 48 h the IC_50_ values were 7.015, 7.316, 8.364, and 5.296 μM, respectively (Fig. 1b). CCK8 assay was also used to assess the cell viability of these four cell lines after treatment with Glaucocalyxin A for 24 and 48 h (Fig. 1c). The CCK8 results further confirmed that MG-63 and HOS cell lines were more susceptive to Glaucocalyxin A; thus we chose these two cell lines in the following experiments with 2.5, 5, 10 μM of Glaucocalyxin A treatment for 24 h. In order to evaluate the safety of the concentrations we used in vitro, we examined the toxicity of Glaucocalyxin A on several human normal cells HEK-293, HUVEC, L02, and BEAS-2B. There were no significant effects on cell viability of HEK-293, HUVEC, L02, and BEAS-2B cells after treatment with the same concentration of Glaucocalyxin A (Fig. 1d, e). These data suggested that Glaucocalyxin A had a selectively inhibitory effect on osteosarcoma cells but not on non-transformed cells.

As shown in Fig. 1f, glaucocalyxin A-treated MG-63 cells became round and broke into fragments. The degree of sloughing of cells was correlated with drug concentrations. To observe the morphological changes of HOS and MG-63 cells in the presence of Glaucocalyxin A for 24 h, we used DAPI staining assay to test if Glaucocalyxin A induced apoptosis in MG-63 and pHOS cells. The result showed that the control cells remained round shaped, whereas cells treated with Glaucocalyxin A presented morphological features of apoptotic chromatin condensation and DNA fragmentation in a dose-dependent manner (Fig. 1g). These results suggested that the inhibitory effect of Glaucocalyxin A on the growth of osteosarcoma cells might be attributed to inducing apoptosis. Annexin V/PI staining assay was used to confirm the pro-apoptotic effect of Glaucocalyxin A. The apoptotic rates of HOS and MG-63 cells were significantly increased by Glaucocalyxin A in a dose-dependent manner, compared with the control group (Fig. 1h). The quantitative analysis for the percentage of apoptotic cells showed that Glaucocalyxin A remarkably induced apoptosis in HOS and MG-63 cells (Fig. 1f). Moreover, we added pan-caspase inhibitor Z-VAD-FMK to demonstrate that the cell death was primarily caspase-dependent apoptosis. The results showed that the pan-caspase inhibitor Z-VAD-FMK reversed the pro-apoptotic effects of Glaucocalyxin A on HOS and MG-63 cells (Supplementary Fig. 1a and b). Our data demonstrated that Glaucocalyxin A induced typical apoptosis in human osteosarcoma cells.

### Glaucocalyxin A induced mitochondria-mediated apoptosis in HOS and MG-63 cells

**Fig. 2:**
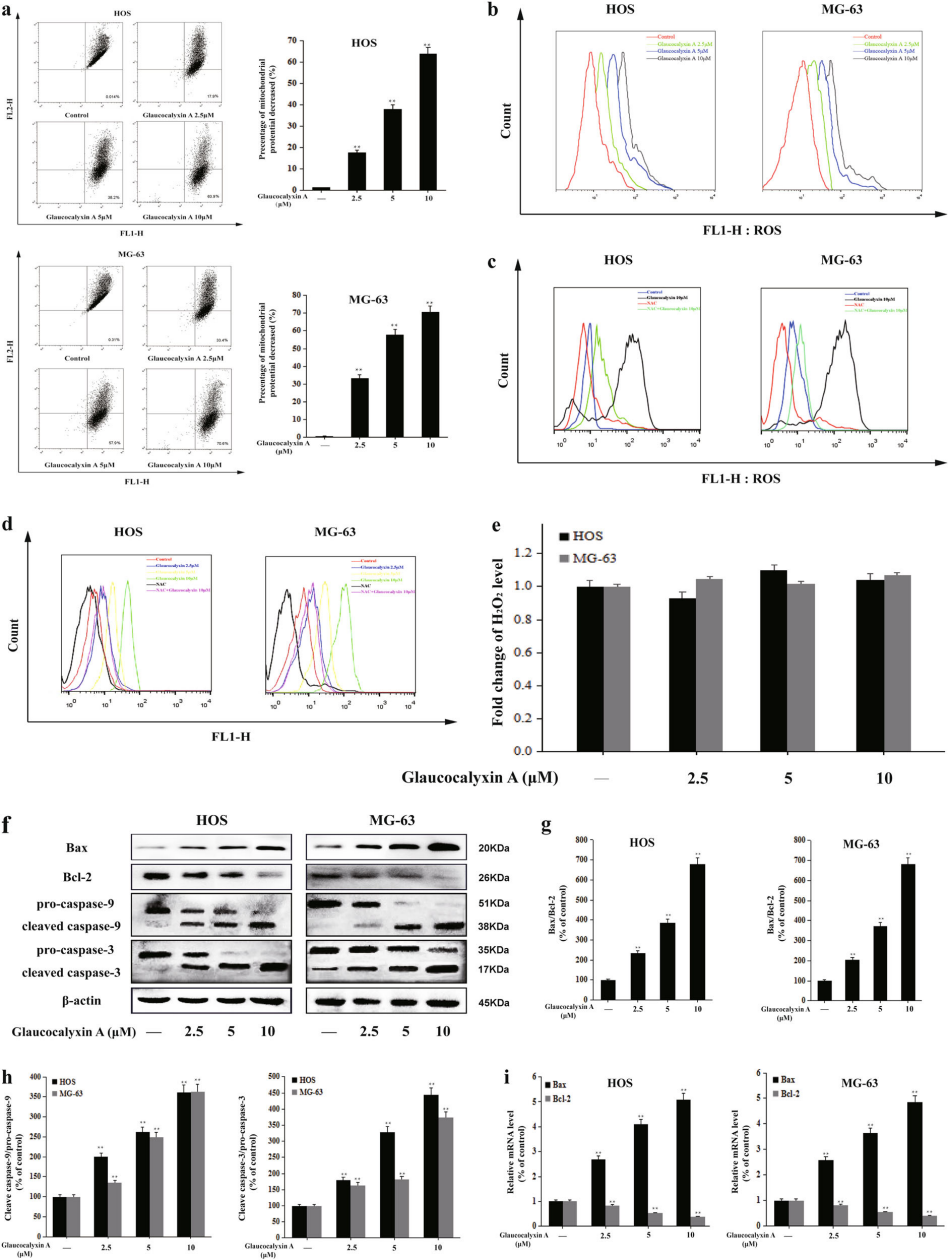
Glaucocalyxin A induced mitochondrial apoptosis in HOS and MG-63 cells. **a** The percentage change of ΔΨm was detected by using JC-1 staining. **b** The generation of intracellular ROS of HOS and MG-63 cells after treatment with Glaucocalyxin A was measured by flow cytometry. **c** Cells were pre-incubated with/without NAC (5 mM) for 2 h, then exposed to 10 μM Glaucocalyxin A for 24 h, followed by ROS measurement. **d** The O_2_^.−^ level of HOS and MG-63 cells after treatment with 2.5, 5, and 10 μM Glaucocalyxin A for 24 h and the cells pre-incubated with/without NAC (5 mM) for 2 h, then exposed to 10 μM Glaucocalyxin A for 24 h. **e** The H_2_O_2_ level of HOS and MG-63 cells after treatment with 2.5, 5, and 10 μM Glaucocalyxin A for 24 h. **f** The protein expression of Bax, Bcl-2, pro-caspase-9, cleaved caspase-9, pro-caspase-3, and cleaved caspase-3 was determined by western blot with β-actin as an internal control. **g** Gray scale analysis was performed to determine the relative ratios of Bax/Bcl-2. The results are shown as means ± SD from three independent experiments. ^**^P < 0.01 compared with the control group. **h** Gray scale analysis was performed to determine the relative ratios of cleaved caspase-9/pro-caspase-9 and cleaved caspase-3/pro-caspase-3. The results are shown as means ± SD from three independent experiments. ^**^P < 0.01 compared with the control group. **i** The mRNA levels of Bax and Bcl-2 were determined by real-time PCR. The results are shown as means ± SD from three independent experiments. ^**^P < 0.01 compared with the control group

The mitochondrial function is important for cell survival. The change of MMP (ΔΨm) is a hallmark of mitochondrial dysfunction in early apoptosis. To further investigate Glaucocalyxin A-induced apoptosis, MMP (ΔΨm) was detected by flow cytometry. The value of average cell MMP dramatically decreased after the treatment with glaucocalyxin A (Fig. 2a). The results suggested that Glaucocalyxin A induced apoptosis by aggravating the mitochondrial dysfunction in osteosarcoma cells.

Oxidative stress is an important factor causing mitochondrial dysfunction^34^. Moreover, reactive oxygen species (ROS) play a key role in cell apoptosis^35^. Therefore, we examined the effect of Glaucocalyxin A on ROS generation in HOS and MG-63 cells. The results showed that Glaucocalyxin A increased the generation of ROS in a concentration-dependent manner (Fig. 2b), suggesting that Glaucocalyxin A triggered the generation of intracellular ROS. We used *N*-acetylcysteine (NAC), an ROS scavenger, to examine the effect of Glaucocalyxin A on the generation of ROS. The results revealed that the level of ROS triggered by Glaucocalyxin A was alleviated by NAC (Fig. 2c). To further determine the kind of ROS overproduced by Glaucocalyxin A, we measured intracellular superoxide anion (O_2_^.−^) and hydrogen peroxide (H_2_O_2_) levels in osteosarcoma cells^36^. Similar with ROS, intracellular O_2_^.−^ level could be elevated by Glaucocalyxin A and reversed by NAC (Fig. 2d), whereas H_2_O_2_ level remained unchanged (Fig. 2e). These results manifested that mainly ROS triggered by glaucocalyxin A was O_2_^.−^.

We detected the levels of the apoptosis-related proteins such as Bcl-2, Bax, cleaved caspase-9, cleaved caspase-3 by western blot. After treatment with Glaucocalyxin A for 24 h, the protein expression of the apoptotic protein Bax increased while the protein expression of the antiapoptotic protein Bcl-2 decreased in a concentration-dependent manner (Fig. 2f). The ratio of Bax/Bcl-2 is an indicator of mitochondrial apoptotic pathway. Our results revealed that the ratio of Bax/Bcl-2 was markedly increased by Glaucocalyxin A (Fig. 2g). Caspase-9 and caspase-3 cleavage were remarkably activated after Glaucocalyxin A treatment (Fig. 2f, h). Besides, the mRNA levels of Bcl-2 and Bax were also regulated by Glaucocalyxin A accordingly (Fig. 2i). All these findings indicated that Glaucocalyxin A induced mitochondrial apoptosis in osteosarcoma cells.

### Glaucocalyxin A induced apoptosis by inhibiting GLI1 nuclear translocation in human osteosarcoma cells

**Fig. 3:**
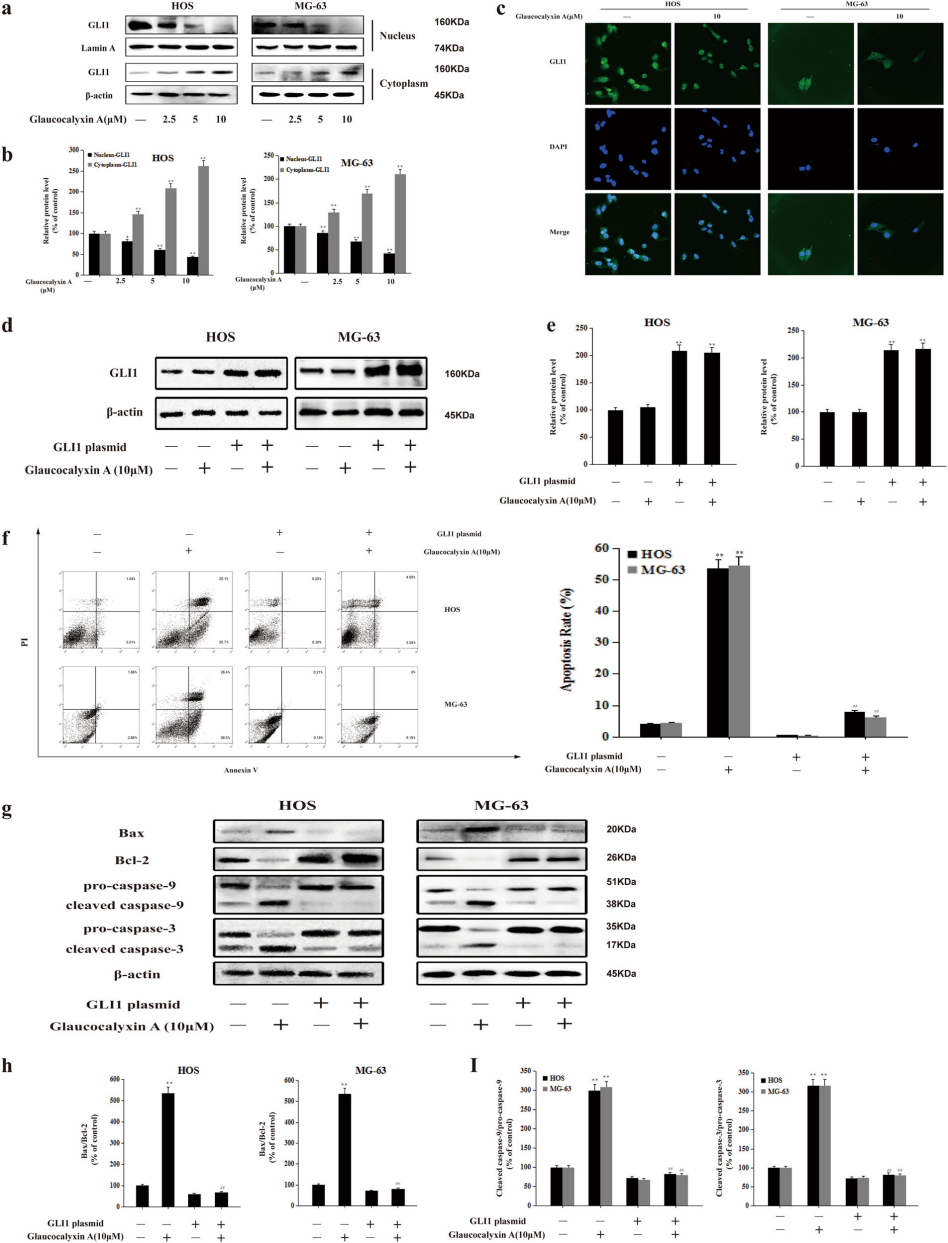
Glaucocalyxin A induced apoptosis by inhibiting GLI1 activation in HOS and MG-63 cells. **a**−**c** HOS and MG-63 cells were treated with or without Glaucocalyxin A (2.5, 5, and 10 μM) for 24 h. **a** The nuclear and cytoplasmic expression of GLI1 in HOS and MG-63 cells was detected by western blot analysis. Lamin A and β-actin were used as nuclear and cytoplasmic markers, respectively. **b** Gray scale analysis was performed to determine the relative ratio of GLI1 protein expression. The results are shown as means ± SD from three independent experiments. ^*^P < 0.05 and ^**^P < 0.01 compared with the control group. **c** The nuclear translocation of GLI1 in HOS and MG-63 cells was detected by immunofluorescence staining (×400). **d**−**k** HOS and MG-63 cells were transfected with GLI1 plasmid followed by with or without Glaucocalyxin A (10 μM) for 24 h. **d** The protein expression of GLI1 in HOS and MG-63 cells was assessed by western blot. β-actin was used as an internal control. **e** Gray scale analysis was performed to determine the relative ratios of GLI1. The results are shown as means ± SD from three independent experiments. ^**^P < 0.01 compared with the control group. **f** The apoptotic rates of HOS and MG-63 cells were detected by Annexin V/PI double-staining assay. The results are shown as means ± SD from three independent experiments. ^**^P < 0.01 compared with the control group; ^##^*P* < 0.01 compared with the Glaucocalyxin A (10 μM) group. **g** The protein expression of Bax, Bcl-2 pro-caspase-9, cleaved caspase-9, pro-caspase-3, and cleaved caspase-3 was assessed by western blot. β-actin was used as an internal control. **h**, **i** Gray scale analysis was performed to determine the relative ratios of Bax/Bcl-2, cleaved caspase-9/pro-caspase-9, and cleaved caspase-3/pro-caspase-3. The results are shown as means ± SD from three independent experiments. ^**^P < 0.01 compared with the control group; ^##^*P* < 0.01 compared with the Glaucocalyxin A (10 μM) group

GLI is in charge of regulating normal physiological activities and many diseases such as cancer. GLI activation is crucial in several stages of tumorigenesis. When it is inhibited, the number of apoptotic cells significantly increased^18,26,27^. The activation of GLI promoted the development of osteosarcoma^37,38^. Here we investigated the inhibitory effect of Glaucocalyxin A on the activation of GLI1 in human osteosarcoma cells. Western blot analysis showed that Glaucocalyxin A decreased the nuclear expression of GLI1, while it increased the cytoplasmic expression of GLI1 in a concentration-dependent manner (Fig. 3a, b). This result was further confirmed by the immunofluoresence staining. The result showed that 10 μM Glaucocalyxin A inhibited the nuclear translocation of GLI1 (Fig. 3c). We further used GLI1 plasmid to confirm the effect of Glaucocalyxin A on apoptosis in vitro. Western blot analysis showed that GLI1 protein was overexpressed by GLI1 plasmid (Fig. 3d, e). GLI1 plasmid significantly attenuated Glaucocalyxin A-induced apoptosis in MG-63 and HOS cells (Fig. 3f). Moreover, we detected the effects of Glaucocalyxin A on the protein expression of Bax, Bcl-2, cleaved caspase-9, and cleaved caspase-3 after transfection with GLI1 plasmid. In the presence of GLI1 plasmid, the effects of Glaucocalyxin A on the protein expression of apoptosis-related proteins were obviously reversed (Fig. 3g−i). We also used GLI1 siRNA to confirm the effect of Glaucocalyxin A on apoptosis in vitro. As expected, GLI1 siRNA reduced GLI1 protein efficiently (Supplementary Fig. 2a and b). After transfection with GLI1 siRNA, Glaucocalyxin A hardly had effect on apoptosis (Supplementary Fig. 2c and d) and the protein expression of apoptosis-related proteins in osteosarcoma cells (Supplementary Fig. 2e−h). These results suggested that Glaucocalyxin A induced apoptosis by inhibiting nuclear translocation of GLI1.

### Glaucocalyxin A induced apoptosis via inhibiting GLI1 nuclear translocation by regulating PI3K/Akt signaling pathway in human osteosarcoma cells

**Fig. 4:**
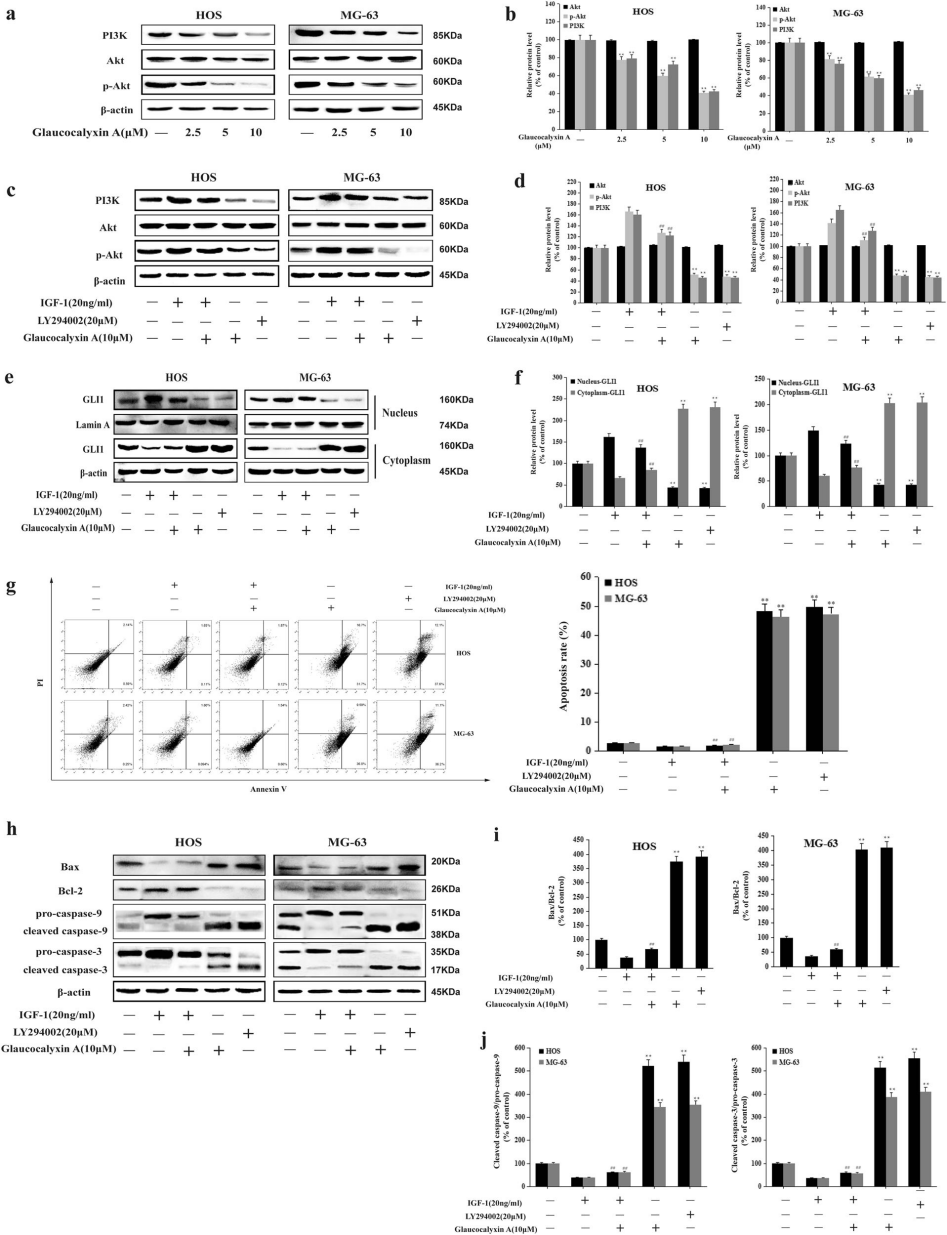
Glaucocalyxin A induced apoptosis via inhibiting GLI1 activation by regulating PI3K/Akt signaling pathway in HOS and MG-63 cells. **a** The protein expression of PI3K, p-Akt, and Akt in HOS and MG-63 cells was assessed by western blot. β-actin was used as an internal control. **b** Gray scale analysis was performed to determine the relative ratios of PI3K, p-Akt, and Akt. The results are shown as means ± SD from three independent experiments. ^**^P < 0.01 compared with control group. **c**−**j** HOS and MG-63 cells were treated with IGF-1 (20 ng/ml) with or without Glaucocalyxin A (10 μM) for 24 h. HOS and MG-63 cells were treated with LY294002 (20 μM) for 24 h. **c** The protein expression of PI3K, p-Akt, and Akt was detected by western blot. β-actin was used as an internal control. **d** Gray scale analysis was performed to determine the relative ratios of PI3K, p-Akt, and Akt. The results are shown as means ± SD from three independent experiments. ^**^P < 0.01 compared with the control group; ^##^*P* < 0.01 compared with the Glaucocalyxin A (10 μM) group. **e** The nuclear and cytoplasmic expression of GLI1 was detected by western blot. Lamin A and β-actin were used as nuclear and cytoplasmic markers, respectively. **f** Gray scale analysis was performed to determine the relative ratio of GLI1 protein expression. The results are shown as means ± SD from three independent experiments. ^**^P < 0.01 compared with the control group; ^##^*P* < 0.01 compared with the Glaucocalyxin A (10 μM) group. **g** Annexin-V/PI staining assay was measured by flow cytometry. The results are shown as means ± SD from three independent experiments. ^**^P < 0.01 compared with the control group; ^##^*P* < 0.01 compared with the Glaucocalyxin A (10 μM) group. **h** The protein expression of Bax, Bcl-2 pro-caspase-9, cleaved caspase-9, pro-caspase-3, and cleaved caspase-3 was assessed by western blot. β-actin was used as an internal control. **i**, **j** Gray scale analysis was performed to determine the relative ratios of Bax/Bcl-2, cleaved caspase-9/pro-caspase-9, and cleaved caspase-3/pro-caspase-3. The results are shown as means ± SD from three independent experiments. ^**^P < 0.01 compared with the control group; ^##^*P* < 0.01 compared with the Glaucocalyxin A (10 μM) group

It has been reported that PI3K/Akt signaling pathway plays a key role in osteosarcoma progression^39^. We investigated the effect of Glaucocalyxin A on PI3K/Akt pathway. Our results showed that the protein expression of PI3K and p-Akt in HOS and MG-63 cells was decreased by Glaucocalyxin A. The protein expression of Akt remained constant after treatment with Glaucocalyxin A (Fig. 4a, b). We further used an activator and an inhibitor of PI3K/Akt signaling pathway, IGF-1 and LY294002, to determine whether Glaucocalyxin A inhibited PI3K/Akt pathway. The inhibitory effect of Glaucocalyxin A on PI3K/Akt signaling pathway was reversed by the treatment with 20 ng/ml IGF-1 (Fig. 4c, d). These results indicated that Glaucocalyxin A inhibited PI3K/Akt signaling pathway.

Recent report has demonstrated that PI3K/Akt pathway can regulate the activation of GLI. We further used IGF-1 and LY294002 to confirm whether Glaucocalyxin A inhibited GLI1 activation by regulating PI3K/Akt pathway. The results showed that 10 μM glaucocalyxin A inhibited the nuclear translocation of GLI1; however, this effect was also withdrawn by IGF-1 (Fig. 4e, f). Moreover, there was no significant difference between the inhibitory effect of Glaucocalyxin A and LY294002 on PI3K/Akt signaling pathway and the nuclear translocation of GLI1 (Fig. 4e, f).

We continued to examine whether Glaucocalyxin A induced apoptosis by regulating PI3K/Akt pathway using IGF-1 and LY294002. The pro-apoptotic effect of Glaucocalyxin A was reversed by the treatment with 20 ng/ml IGF-1 (Fig. 4g). Moreover, the ratio of Bax/Bcl-2 and the cleavage of caspase-9 and caspase-3 increased by Glaucocalyxin A was withdrawn by 20 ng/ml IGF-1 (Fig. 4h−j). There was no significant difference between the pro-apoptotic effect of Glaucocalyxin A and LY294002 (Fig. 4g−j). Moreover, we used PI3K siRNA to confirm the effect of Glaucocalyxin A on PI3K signaling in vitro. PI3K siRNA reduced PI3K protein efficiently and Glaucocalyxin A had little effect on phosphorylation of Akt (Supplementary Fig. 3a and b). After transfection with PI3K siRNA, Glaucocalyxin A hardly had any effect on apoptosis (Supplementary Fig. 3c and d) and the protein expression of apoptosis-related proteins in osteosarcoma cells (Supplementary Fig. 3e−h). Altogether, our results suggested that Glaucocalyxin A induced apoptosis via inhibiting GLI1 nuclear translocation by regulating PI3K/Akt signaling pathway.

### Glaucocalyxin A inhibited tumor growth by inducing apoptosis via inhibiting GLI1 nuclear translocation through regulating PI3K/Akt pathway in vivo

**Fig. 5:**
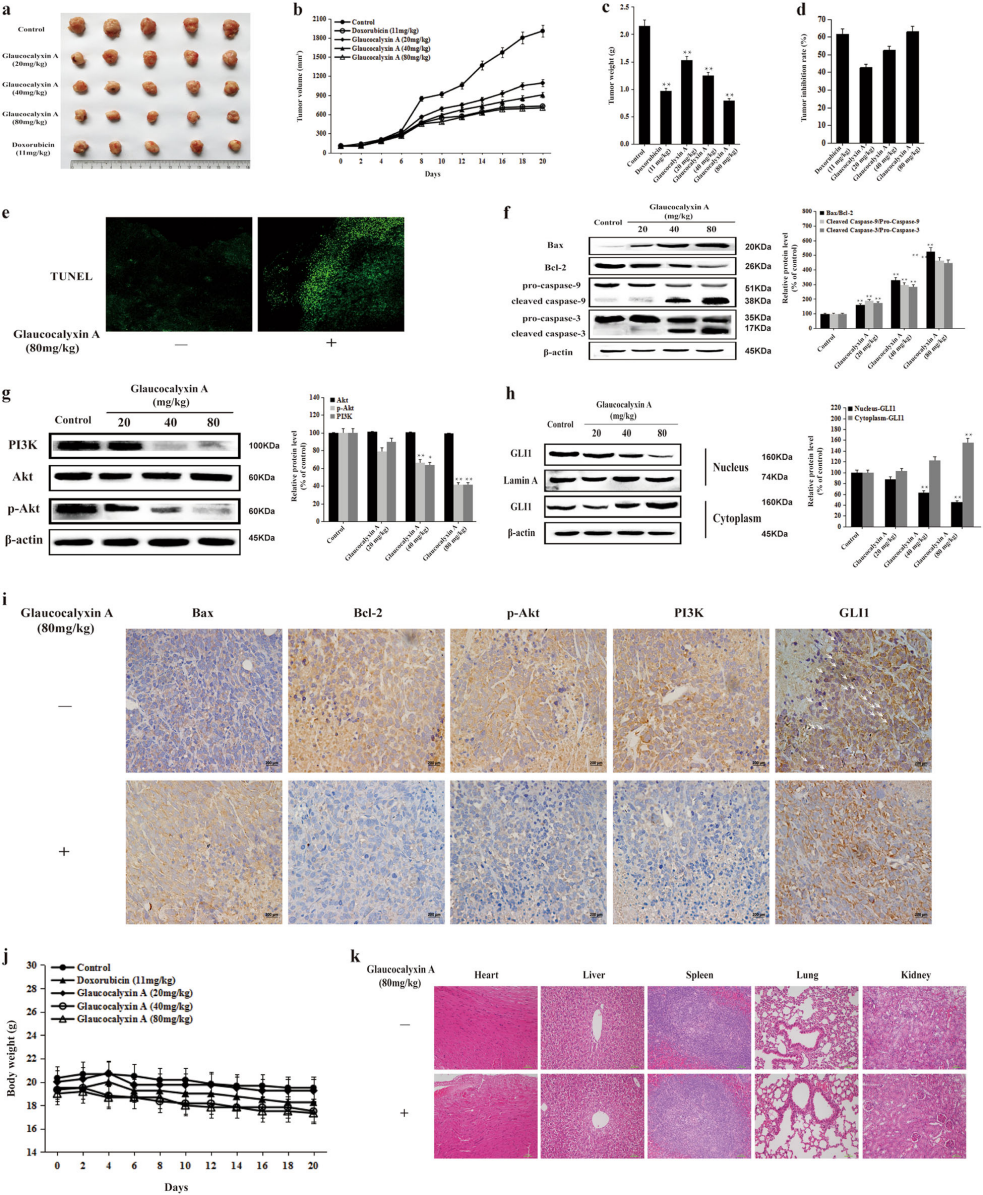
Glaucocalyxin A inhibited tumor growth and induced apoptosis in tumor tissues via inhibiting GLI1 activation through regulating PI3K/Akt pathway in vivo. The nude mice bearing HOS osteosarcoma cells were treated with 11 mg/kg doxorubicin and 20, 40, 80 mg/kg of Glaucocalyxin A every 2 days. Control group was treated with saline. **a** The picture of nude mice xenograft tumors was captured after the treatment for 21 days. **b** Tumor volume of the control, doxorubcin, and Glaucocalyxin A treatment groups was measured and calculated once every 2 days. **c** Weight of tumor in the control, doxorubcin, and Glaucocalyxin A treatment groups. **d** Inhibitory activity of Glaucocalyxin A against HOS xenograft tumor. **e** The DNA damage in tumor tissues was detected by the TUNEL test (×200). **f** The protein expression of Bax, Bcl-2, pro-caspase-9, cleaved caspase-9, pro-caspase-3, and cleaved caspase-3 in tumor tissues was determined by western blot analysis. β-actin was used as the loading control. Gray scale analysis was performed to determine the relative ratios of Bax/Bcl-2, cleaved caspase-9/procaspase-9, cleaved caspase-3/pro-caspase-3. The results are shown as means ± SD from three independent experiments. ^**^P < 0.01 compared with control group. **g** The protein expression of PI3K, p-Akt, and Akt in tumor tissues was detected by western blot analysis with β-actin as the loading control. Gray scale analysis was performed to determine the relative ratios of PI3K, p-Akt, and Akt. The results are shown as means ± SD from three independent experiments. ^*^P < 0.05 and ^**^P < 0.01 compared with the control group. **h** The nuclear and cytoplasmic expression of GLI1 in tumor tissues was determined by western blot analysis. Lamin A and β-actin were used as nuclear and cytoplasmic markers, respectively. Gray scale analysis was performed to determine the relative ratio of GLI1 protein expression. The results are shown as means ± SD from three independent experiments. ^**^P < 0.01 compared with the control group. **i** The protein expression of Bax, Bcl-2, p-Akt, PI3K, and GLI1 was detected by immunohistochemistry in tumor tissues (×400). **j** Body weight was measured every 2 days. **k** The major organs (heart, liver, spleen, lung, and kidney) were analyzed by H&E staining (×200)

To evaluate the anticancer effect of Glaucocalyxin A in vivo, we established a murine xenograft model bearing HOS cells in this study. After 21 days of experiment, there were significant differences in tumor volume across groups between the treatment groups and the control group. Glaucocalyxin A remarkably inhibited tumor growth and decreased the tumor volume (Fig. 5a−d). To prove our previous conclusion in vitro, TUNEL staining assay was used to investigate the pro-apoptotic effect of Glaucocalyxin A on tumors in xenografts with osteosarcoma. We found obviously increased number of TUNEL-positive cells in the tumor tissues from Glaucocalyxin A-treated mice compared with the control group (Fig. 5e). Furthermore, Glaucocalyxin A dose-dependently increased the ratio of Bax/Bcl-2 and the cleavage of caspase-3 and caspase-9 in tumor tissues (Fig. 5f). Glaucocalyxin A also inhibited the protein expression of PI3K, p-Akt, and nuclear translocation of GLI in the tumor tissues (Fig. 5g, h). The results were further confirmed by immunohistochemistry staining analysis (Fig. 5i). Our data demonstrated that Glaucocalyxin A inhibited tumor growth by inducing apoptosis via inhibiting GLI nuclear translocation through regulating PI3K/Akt signaling pathway in vivo.

Though Glaucocalyxin A inhibited the growth of osteosarcoma in vivo, its potential toxicity must be assessed comprehensively. During this study (21 days), we noticed that the body weight of mice in the control and glaucocalyxin A groups was not significantly different (Fig. 5j). We also found no obvious change in major organs between the control and Glaucocalyxin A-treated groups (Fig. 5k). Moreover, hematological parameters were normal in 80 mg/kg of Glaucocalyxin A-treated mice (Table 1). The results indicated that 80 mg/kg of Glaucocalyxin A exerted anti-tumor activity without any toxicity in vivo*.* All our results above demonstrated that Glaucocalyxin A inhibited tumor growth by inducing apoptosis via inhibiting GLI1 nuclear translocation through regulating PI3K/Akt pathway in human osteosarcoma.

**Table 1:** Effects of Glaucocalyxin A on hematology indices in nude mice

Hematological parameters	Control	Glaucocalyxin A			Standard
		20 mg/kg	40 mg/kg	80 mg/kg	
White blood cells (×10^3^/ml)	7.11 ± 0.81	5.80 ± 0.65	6.94 ± 0.50	6.79 ± 0.98	4.5–9
Red blood cells (×10^6^/blood cell)	8.71 ± 0.43	8.79 ± 0.43	8.96 ± 0.35	9.04 ± 0.17	7.51–9.66
Hemoglobin (g/dl)	14.42 ± 0.59	14.02 ± 0.60	14.08 ± 0.43	13.87 ± 0.30	12.8–16.1
Hematocrit (%)	43.02 ± 2.41	43.83 ± 3.24	44.63 ± 2.40	44.25 ± 0.71	34–50
Mean corpuscular volume (fl)	44.98 ± 1.04	46.40 ± 3.38	45.77 ± 2.82	44.90 ± 1.40	41–60
Mean corpuscular hemoglobin (pg)	14.43 ± 0.27	14.98 ± 0.36	14.60 ± 0.43	14.92 ± 0.39	13–19
Lymphocytes (%)	56.78 ± 2.38	62.00 ± 3.43	56.00 ± 1.97	69.85 ± 6.04	49–82
Monocytes (%)	3.58 ± 0.32	3.13 ± 0.83	2.52 ± 0.32	4.12 ± 0.54	2–8
Eosinophils (%)	2.17 ± 0.43	2.03 ± 0.26	2.57 ± 0.25	2.30 ± 0.30	0–3
Basophils (%)	0.52 ± 0.18	0.57 ± 0.15	0.62 ± 0.29	0.55 ± 0.13	0–3
Platelet	618.17 ± 138.49	673.67 ± 62.80	381.33 ± 52.70	259.50 ± 48.83	115–1037

Each data point represents the mean ± SD of six mice.

*P* < 0.05 versus basal levels of the control group.

## Discussion

Osteosarcoma, the primary bone cancer with high incidence, will always cause death for metastatic disease unless treated by surgery and effective multidrug chemotherapy^4^. It has been reported that high-dose methotrexate, doxorubicin, and cisplatin, with some regimens incorporating ifosfamide, seems to be common methods for treatment^40^. Although we get advances in surgery and targeted therapy for the malignancy nowadays, most patients still have a higher recurrence rate as well as a lower survival rate. Therefore, it is indispensable to develop more effective and less toxic drugs for the treatment of osteosarcoma. In this study, we investigated the anticancer effect of Glaucocalyxin A on osteosarcoma and illustrated the underlying mechanisms. Our results demonstrated that Glaucocalyxin A induced apoptosis in osteosarcoma by inhibiting GLI1 via regulating PI3K/Akt signaling pathway in vitro and in vivo.

Apoptosis, a typical programmed cell death with distinct biochemical and genetic pathways in normal tissues, plays an important role in the development and homeostasis^41^. It is responsible for the elimination of cells that threaten the survival and unnecessary cells to maintain the normal balance between cell survival and cell death in mammals^42,43^. In tumors, apoptosis is caused by caspases through targeting cysteine aspartyl^44,45^. Bax, the pro-apoptotic member of the Bcl-2 family, forms ion channels directly causing mitochondria to release cytochrome c^46^, and then activates caspase-9 and caspase-3, which induces apoptosis in the end^47^. In the present study, the apoptotic rates of HOS and MG-63 cells were both remarkably increased by Glaucocalyxin A, suggesting that Glaucocalyxin A induced apoptosis in osteosarcoma. We also found that Glaucocalyxin A induced mitochondrial apoptosis by increasing Bax/Bcl-2 ratio, loss of mitochondrial membrane potential (ΔΨm), triggering ROS generation, and inducing caspase-9 and caspase-3 cleavage in HOS and MG-63 cells. These results demonstrated that Glaucocalyxin A induced mitochondrial apoptosis in osteosarcoma.

The transcription factor GLI is critically important in the Hedgehog signaling pathway. GLI is overexpressed and activated in a variety of cancers, regulating lots of cellular processes, including apoptosis and proliferation^48,49^. There is a study reported that GLI is highly expressed in lung cancer tissue, but not in normal lung tissue via in situ hybridization^50^. Inhibiting GLI can induce apoptosis in cervical cancer stem cells by modulating the transcription of the target genes^51^. The report has documented that GLI also is a therapeutic option for B-cell chronic lymphocytic leukemia^52^. Moreover, GLI inhibitor has a pro-apoptotic effect on myeloid leukemia cells and hepatocellular carcinoma cells by changing the morphogenesis of apoptosis and activating caspase-3^53,54^. The activation of GLI1, via gene amplification including upregulating Bcl-2, has been implicated in the initiation and progression of multiple cancers^55^. Bcl-2, an anti-apoptosis oncogene, is reported as a known transcriptional target of GLI1^56,57^, and can be regulated by GLI activation in cancer^56^. The recent study showed that SUFU-mediated suppression of GLI activity was controlled by a BH3 sequence-dependent interaction between SUFU and three prosurvival Bcl-2 family members such as Bcl-2 and Bcl-xL^58^. In other models, GANT61 inhibition of GLI transcription has been shown to inhibit several cell activities including Bcl-2^59^. Moreover, it has been reported that GLI1 maintained cell survival by binding the promoter regions and facilitating transcription of Bcl-2 genes. Cyclopamine blocked the growth of colorectal cancer SW116 cells by modulating target Bcl-2 family genes of GLI1 including Bcl-2 and Bax in vitro^57^. Therefore, targeting GLI activation may be an attractive strategy for the cancer treatment. It is also reported that GLI signaling is active and regulates the target genes in osteosarcoma cells. Inhibition of GLI is capable to prevent the progression of osteosarcoma^60^. In our study, we demonstrated that Glaucocalyxin A inhibited the nuclear translocation of GLI in osteosarcoma cells HOS and MG-63.

The activation of PI3K/Akt pathway can promote the development of various human cancers such as breast cancer, lung cancer, melanoma, and lymphoma^13^. PI3K/Akt pathway plays a crucial role in multiple processes of cancer, such as apoptosis, proliferation, metastasis, by modulating many downstream transcription factors^61^. It has been demonstrated that inhibiting PI3K/Akt pathway can induce dramatic apoptosis of osteosarcoma^62,63^. Increasing evidence has suggested that GLI protein can be modulated directly and indirectly by proliferative and oncogenic inputs, in addition or independent of upstream Hedgehog signaling^64^. There is a recent study reporting the regulatory effect of PI3K/Akt pathway on GLI activation^29^, demonstrating that GLI1 and GLI2 were activated by PI3K/Akt pathway. PI3K-Akt cascade is reported to maintain the stabilization of GLI1 protein as well^65^. Thus, our significant attention was focused on the nonclassical GLI1 activation regulated by PI3K/Akt pathway. Our results showed that Glaucocalyxin A reduced the protein expression of PI3K and phosphorylated-Akt in HOS and MG-63 cells after treatment with Glaucocalyxin A. The inhibitory effect of Glaucocalyxin A on the activation of GLI was confirmed by PI3K activator (IGF-1) and a PI3K inhibitor (LY294002). Furthermore, Glaucocalyxin A-induced apoptosis was reversed by PI3K activator (IGF-1). The effect of Glaucocalyxin A on the expression of apoptosis-related proteins was also withdrawn by IGF-1. These results demonstrated that Glaucocalyxin A induced apoptosis by inhibiting nuclear translocation of GLI through modulating PI3K/Akt signaling pathway.

The in vitro study showed that Glaucocalyxin A induced mitochondrial apoptosis pathway with increasing Bax/Bcl-2 ratio, reducing MMP, activation of caspase-9 and caspase-3. The results also indicated that apoptosis induced by Glaucocalyxin A in osteosarcoma cells was not cell specific. Moreover, our study first demonstrated that Glaucocalyxin A possessed the pro-apoptotic effect by inhibiting GLI via regulating PI3K/Akt pathway in osteosarcoma. Our further study documented that Glaucocalyxin A exerted the antitumor effect in vivo through inducing apoptosis in tumors. The standard toxicology studies suggested that Glaucocalyxin A may exhibit anticancer effect without obvious toxicity in vivo.

In conclusion, as a natural compound from Chinese herb medicine, Glaucocalyxin A induced apoptosis and inhibited tumor growth by inhibiting nuclear translocation of GLI1 via regulating PI3K/Akt signaling pathway in osteosarcoma cells and in xenograft tumor model. Therefore, our present study suggested that glaucocalyxin A might be a promising agent against human osteosarcoma for its good anticancer efficiency and high safety.

## Materials and methods

### Materials

Glaucocalyxin A is a white amorphous powder with purity of more than 98%, the molecular formula is C_20_H_28_O_4_ with a molecular weight of 332.437 Da, which was purchased from Cheng Du Purechem-Standard Co., Ltd (Sichuan Province, China). Glaucocalyxin A was dissolved in dimethyl sulfoxide (DMSO, Sigma, USA) and DMSO-treated cells were used as a vehicle control. Penicillin, streptomycin, minimum essential medium and McCoy’s 5A (Modified) medium were obtained from Thermo Fisher Scientific (USA). Fetal bovine serum (FBS) was provided by Gibco Life Technologies (New York, USA). MTT [3-(4, 5-dimethylthiazol-2-yl)-2, 5-diphenytetrazoliumbromide] obtained from Sigma Aldrich (USA) was dissolved in 0.01 M PBS. Antibodies of caspase-3, caspase-9, Bax, Bcl-2, Akt, p-Akt, GLI1, PI3K, and β-actin were obtained from Cell Signaling Technology (USA) and were used at a ratio of 1:1000. The caspase inhibitor Z-VAD-FMK was purchased from Beyotime Institute of Biotechnology (Nanjing, China).

### Cell culture

The human osteosarcoma cell lines MG-63, U-2OS, HOS, and Saos-2 and the human normal cells HEK-293, HUVEC, L02, and BEAS-2B were obtained from Cell Bank of Shanghai Institute of Biochemistry and Cell Biology, Chinese Academy of Sciences. The U2OS and Saos-2 cells were cultured in McCoy’s 5A (Modified) medium (Thermo Fisher Scientific, Waltham, MA, USA) with 15% FBS (Gibco; Thermo Fisher Scientific), while the MG-63 and HOS cells were cultured in minimum essential medium (Gibco; Thermo Fisher Scientific) with 10% FBS, containing 100 units/ml penicillin and 100 μg/ml streptomycin. The HEK-293, HUVEC, and BEAS-2B cells were cultured in Dulbecco’s minimum essential medium (Gibco; Thermo Fisher Scientific) with 10% FBS, and L02 was cultured in Roswell Park Memorial Institute-1640 (Gibco; Thermo Fisher Scientific) with 10% FBS, with 100 units/ml penicillin and 100 μg/ml streptomycin. All the cells were cultured at 37 °C in a humidified incubator with 5% CO_2_.

### MTT assay

Cell viability was determined using an MTT colorimetric assay. Cells were seeded onto 96-well plates with 1×10^4^/well in 100 μl culture medium. The cells were incubated in a cell incubator for 24 h and then exposed to drug at the concentrations of 0, 0.05, 0.1, 0.5, 1, 2, 5, 10, 20, and 50 μmol/l glaucocalyxin A for the indicated time points, and the control cells were treated with 0.5% DMSO. Subsequently, 10 μl MTT solution (5 mg/ml in PBS) was added and incubated for 4 h. Supernatants were removed and 100 μl/well DMSO was added at about 25 °C to dissolve formazan crystals. The optical absorbance was recorded at 492 nm by a Thermo Multiskan Mk3 Microplate Reader. The cell growth inhibitory effects were calculated by the following equation: cell viability (%) = (Atreatment/Acontrol) × 100%. A cell growth inhibition curve was generated by plotting cell growth inhibition against drug concentration, and the half-maximal inhibitory concentration (IC_50_) was determined using GraphPad Prism 6 software (GraphPad Software, Inc., La Jolla, CA, USA).

### CCK8 assay

The CCK-8 detection kit (Beyotime Institute of Biotechnology, Nantong, China) was used to measure cell viability according to the manufacturer’s protocol. Cells were seeded onto 96-well plates (5×10^3^ cells per well). After 24 h, cells were treated with different concentrations of Glaucocalyxin A. The cells were cultured respectively for 24 and 48 h. Subsequently, CCK-8 solution was added to each well, and incubated at 37 °C for an additional 3 h. The viable cells were counted by absorbance measurements with a monochromator microplate reader at a wavelength of 450 nm. The optical density value was reported as the percentage of cell viability in relation to the control group (set as 100%).

### Annexin V-FITC/ propidium iodide staining

The apoptosis was analyzed using Annexin V-FITC/propidium iodide (PI) dual staining. HOS and MG-63 osteosarcoma cells were harvested after treatment with Glaucocalyxin A at concentrations of 2.5, 5, and 10 μM for 24 h and stained with Annexin V-FITC/PI Cell Apoptosis Detection Kit (KeyGen Biotech, Nanjing, China) according to the manufacturer’s protocol. The apoptosis rates of the cells were then analyzed by a flow cytometer (BD Biosciences, San Jose, CA, USA).

### Cell morphological assessment

HOS and MG-63 cells were plated onto six-well plates and treated with 2.5, 5, and 10 μM Glaucocalyxin A for 24 h. The cultured cells were observed by the inverted light microscope (Nikon, Chiyodaku, Tokyo, Japan). Cell nucleus was visualized after DNA staining with the fluorescent dye 4′-6-Diamidino-2-phenylindole (DAPI) (KeyGen Biotech, Nanjing, China). Cells were incubated in the dark for 10 min and washed with PBS twice. The nuclear morphology was observed using fluorescence microscope (Nikon, Chiyodaku, Tokyo, Japan).

### Western blot analysis

Osteosarcoma cells were treated with 2.5, 5, and 10 μM Glaucocalyxin A for 24 h, and lysed in RIPA Lysis Buffer (Beyotime Institute of Biotechnology, China). The lysates were then centrifuged at 12,000 rpm for 15 min at 4 °C. The concentrations of the total proteins were determined using the BCA assay by Varioskan spectrofluorometer (Beyotime Institute of Biotechnology, China). The protein was separated with 12% SDS-PAGE gel, transferred onto the PVDF membranes (Millipore, Billerica, MA) and then incubated with specific antibodies overnight at 4 °C followed by incubation with secondary antibodies (Cell Signaling Technology, USA) for 60 min at room temperature. The protein bands were detected using Bioshine ChemiQ series 4800 Mini System (Bioshine, Shanghai, China).

### Quantitative real-time PCR analysis

Total RNA was isolated using the TriPure solution (Takara Bio, Inc., Otsu, Shiga, Japan) after glaucocalyxin A treatment, and then cDNA templates were generated by reverse transcription reaction using Primescript reverse transcriptase (Takara Bio, Inc.) according to the manufacturer’s instructions. Then, the cDNAs were used as templates for determining the expression of related genes by quantitative real-time PCR. Each assay was done in triplicate.

### Measurement of reactive oxygen species and superoxide anions (O_2_^.−^) level

The generation of intracellular ROS and superoxide anions (O_2_^.−^) level were detected using fluorescent dye 2′, 7′-dichlorfluorescein-diacetate (DCFH-DA) (KeyGen Biotechnology, China), DHE (S0063; Beyotime Institute of Biotechnology, Nantong, China), respectively, according to the manufacturer’s protocols. The samples were pretreated with or without 5 mM NAC (S0077; Beyotime Institute of Biotechnology, Nantong, China) for 2 h before cells were treated with 2.5, 5, and 10 μM of Glaucocalyxin A for 24 h. Cells were collected and incubated with the corresponding dye in serum-free medium in 5% CO_2_ at 37 °C for 20 min. After washing by serum-free medium twice, the fluorescence intensity was measured by FACSCalibur flow cytometry (Becton–Dickinson) at Ex./Em. −488/525 nm.

### Hydrogen peroxide (H_2_O_2_) assay

The content of H_2_O_2_ in treated cells was analyzed with a hydrogen peroxide assay kit (S0038; Beyotime Institute of Biotechnology, Nantong, China) according to the manufacturer’s instructions. In brief, cells were harvested, lysed, and centrifuged at 12,000 × *g* for 5 min. Then, test tubes containing 50 μl of supernatants and 100 μl of test solution were placed at room temperature for 30 min, and were measured immediately at a wavelength of 560 nm.

### Measurement of mitochondrial membrane potential

Quantitative changes of MMP were determined by flow cytometry using 5,5′,6,6′-Tetrachloro-1,1′,3,3′-tetraethyl-imidacarbocyanine iodide (JC-1) Apoptosis Detection Kit (Beyotime Institute of Biotechnology, China). Briefly, after harvesting glaucocalyxin A-treated cells, they were incubated with JC-1 for 20 min at 37 °C. Then the cells were washed with cold buffer, resuspended and analyzed by flow cytometry (FACSCalibur, Becton-Dickinson).

### Immunofluorescence

HOS and MG-63 cells were treated with Glaucocalyxin A (10 μM) for 24 h and then harvested. The cells were fixed with 4% formaldehyde, permeabilized with 0.2% Triton^®^ X-100. Then cells were incubated with primary antibody at 37 °C for 1 h and overnight at 4 °C, and added the secondary antibody (Cell Signaling Technology, USA) at 25 °C for 1 h. The cells were washed with PBS, incubated with DAPI staining solution for 5 min. After washing with PBS, samples were observed with a confocal laser scanning microscope (Fluoview FV1000, Olympus, Tokyo, Japan).

### Transfection of GLI1 plasmid, GLI1 siRNA, and PI3K siRNA

HOS and MG-63 cells were plated in six-well plates with fresh medium. GLI1-plasmid, GLI1 siRNA, and PI3K siRNA transfections were performed according to the manufacturer’s instructions of Lipofectamine 2000 reagent (Invitrogen, Carlsbad, CA, USA). After that, cells were exposed to Glaucocalyxin A and harvested for further experiments.

### Antitumor effects in nude mice

Male BALB/c nude mice (35–40-day-old), weighing 18–22 g, were purchased from the Comparative Medicine Centre of Yangzhou University. The animal study was carried out according to National Institutes of Health regulations and approved by the Institutional Animal Care and Use Committee. The mice were maintained in a pathogen-free environment (21 ± 2 °C and 45 ± 10% humidity) on a 12 h light and 12 h dark cycle with food and water supplied freely during the entire experiment. On day 1, 5×10^6^ HOS cells suspended in 100 μl PBS were subcutaneously inoculated in the right flank of each nude mice. After 10–12 days, when tumor sizes reached around 80–150 mm^3^, nude mice with similar tumor volume were randomly assigned to four groups (with six nude mice/group). Glaucocalyxin A (20, 40, 80 mg/kg) groups received intraperitoneal injection of 20, 40, 80 mg/kg/2 days respectively. The control group was administered saline. Tumor volume (TV) was measured daily to observe dynamic changes in tumor growth and calculated according to the formula: *TV* (mm^3^) = 0.5 × *d*^2^ × *D*, where *d* and *D* are the shortest and the longest diameters, respectively. At the end of 21 days, all nude mice were sacrificed, and the tumor tissues were removed and measured. The major organs of the mice were removed for the toxicity assessment.

### TUNEL assay

The terminal deoxynucleotidyl transferase-mediated dUTP nick-end labeling (TUNEL) assay was used to analyze the apoptosis induction in the tumor tissues. It was carried out on xenograft murine model treated as previously described using an in situ cell death detection kit following the manufacturer’s protocol. The slides were photographed under an Olympus FV1000 confocal microscope.

### Immunohistochemistry

The protein expression of Bax, Bcl-2, p-Akt, GLI1, PI3K of the tumor tissues was assessed as described in the previous study^66^.

### Statistical analysis

All data were shown as mean ± standard deviation (SD) from at least three independent experiments, each in triplicate samples for individual treatment or dosage. Statistical analyses were performed using one-way ANOVA analysis of variance with Dunnett’s test. All comparisons are made relative to untreated controls and significance of difference is indicated as *P < 0.05 and **P < 0.01.

## Electronic supplementary material

Supplement Figure 1

Supplement Figure 2

Supplement Figure 3

Supplementary figure legends

## References

[1] Bielack SS, Hecker-Nolting S, Blattmann C, Kager L (2016). Advances in the management of osteosarcoma. F1000Res..

[2] Bielack SS (2002). Prognostic factors in high-grade osteosarcoma of the extremities or trunk: an analysis of 1,702 patients treated on neoadjuvant cooperative osteosarcoma study group protocols. J. Clin. Oncol.: Off. J. Am. Soc. Clin. Oncol..

[3] Bielack S (2013). Controversies in childhood osteosarcoma. Minerva Pediatr..

[4] Ferrari S, Serra M (2015). An update on chemotherapy for osteosarcoma. Expert Opin. Pharmacother..

[5] Kager L (2003). Primary metastatic osteosarcoma: presentation and outcome of patients treated on neoadjuvant Cooperative Osteosarcoma Study Group protocols. J. Clin. Oncol.: Off. J. Am. Soc. Clin. Oncol..

[6] Zhuo B (2015). PI3K/Akt signaling mediated Hexokinase-2 expression inhibits cell apoptosis and promotes tumor growth in pediatric osteosarcoma. Biochem. Biophys. Res. Commun..

[7] Safdari Y, Khalili M, Ebrahimzadeh MA, Yazdani Y, Farajnia S (2015). Natural inhibitors of PI3K/AKT signaling in breast cancer: emphasis on newly-discovered molecular mechanisms of action. Pharmacol. Res..

[8] Perez-Ramirez C, Canadas-Garre M, Molina MA, Faus-Dader MJ, Calleja-Hernandez MA (2015). PTEN and PI3K/AKT in non-small-cell lung cancer. Pharmacogenomics.

[9] Liu S (2017). Effects of miR-145-5p through NRAS on the cell proliferation, apoptosis, migration, and invasion in melanoma by inhibiting MAPK and PI3K/AKT pathways. Cancer Med..

[10] Maurya AK, Vinayak M (2017). PI-103 attenuates PI3K-AKT signaling and induces apoptosis in murine T-cell lymphoma. Leuk. Lymphoma.

[11] Zhao Y (2015). LYG-202 exerts antitumor effect on PI3K/Akt signaling pathway in human breast cancer cells. Apoptosis.

[12] Zu K (2013). Protein expression of PTEN, insulin-like growth factor I receptor (IGF-IR), and lethal prostate cancer: a prospective study. Cancer Epidemiol. Biomarkers Prev..

[13] Toren P, Zoubeidi A (2014). Targeting the PI3K/Akt pathway in prostate cancer: challenges and opportunities (Review). Int. J. Oncol..

[14] Chang F (2003). Involvement of PI3K/Akt pathway in cell cycle progression, apoptosis, and neoplastic transformation: a target for cancer chemotherapy. Leukemia.

[15] Anilkumar U. & Prehn J. H. M. Anti-apoptotic BCL-2 family proteins in acute neural injury. *Front. Cell. Neurosci.* **8**, 281 (2014). 10.3389/fncel.2014.00281 PMC4179715 25324720

[16] Zhang J, Yu XH, Yan YG, Wang C, Wang WJ (2015). PI3K/Akt signaling in osteosarcoma. Clin. Chim. Acta.

[17] Jin S (2007). Grifolin induces apoptosis via inhibition of PI3K/AKT signalling pathway in human osteosarcoma cells. Apoptosis.

[18] Varjosalo M, Taipale J (2008). Hedgehog: functions and mechanisms. Gene Dev..

[19] Merchant AA, Matsui W (2010). Targeting hedgehog—a cancer stem cell pathway. Clin. Cancer Res..

[20] Kalderon D (2000). Transducing the Hedgehog signal. Cell.

[21] Hooper JE, Scott MP (2005). Communicating with Hedgehogs. Nat. Rev. Mol. Cell Bio..

[22] Kasper M, Regi G, Frischauf AM, Aberger F (2006). GLI transcription factors: mediators of oncogenic Hedgehog signalling. Eur. J. Cancer.

[23] Ruiz i Altaba A, Mas C, Stecca B (2007). The Gli code: an information nexus regulating cell fate, stemness and cancer. Trends Cell Biol..

[24] Yang L, Xie G, Fan Q, Xie J (2010). Activation of the hedgehog-signaling pathway in human cancer and the clinical implications. Oncogene.

[25] Scales SJ, de Sauvage FJ (2009). Mechanisms of Hedgehog pathway activation in cancer and implications for therapy. Trends Pharmacol. Sci..

[26] Chan LH (2014). Hedgehog signaling induces osteosarcoma development through Yap1 and H19 overexpression. Oncogene.

[27] Amakye D, Jagani Z, Dorsch M (2013). Unraveling the therapeutic potential of the Hedgehog pathway in cancer. Nat. Med..

[28] Lin ZX (2016). Suppression of GLI sensitizes medulloblastoma cells to mitochondria-mediated apoptosis. J. Cancer Res. Clin..

[29] Zhou JC (2016). Non-canonical GLI1/2 activation by PI3K/AKT signaling in renal cell carcinoma: a novel potential therapeutic target. Cancer Lett..

[30] Kebenko M (2015). ErbB2 signaling activates the Hedgehog pathway via PI3K-Akt in human esophageal adenocarcinoma: identification of novel targets for concerted therapy concepts. Cell Signal..

[31] Gao LW, Zhang JA, Yang WH, Wang B, Wang JW (2011). Glaucocalyxin A induces apoptosis in human leukemia HL-60 cells through mitochondria-mediated death pathway. Toxicol. Vitr..

[32] Xiao X (2013). Glaucocalyxin A, a negative Akt regulator, specifically induces apoptosis in human brain glioblastoma U87MG cells. Acta Biochim. Biophys. Sin..

[33] Li M, Jiang XG, Gu ZL, Zhang ZB (2013). Glaucocalyxin A activates FasL and induces apoptosis through activation of the JNK pathway in human breast cancer cells. Asian Pac. J. Cancer Prev..

[34] Jo S (2017). Myricetin induces apoptosis of human anaplastic thyroid cancer cells via mitochondria dysfunction. Anticancer Res..

[35] Liu H (2017). Glibenclamide, a diabetic drug, prevents acute radiation-induced liver injury of mice via up-regulating intracellular ROS and subsequently activating Akt-NF-kappa B pathway. Oncotarget.

[36] Forman HJ (2015). Even free radicals should follow some rules: a guide to free radical research terminology and methodology. Free Radic. Bio. Med..

[37] Lo WW (2014). Involvement and targeted intervention of dysregulated Hedgehog signaling in osteosarcoma. Cancer-Am. Cancer Soc..

[38] Schreiber RD, Old LJ, Smyth MJ (2011). Cancer immunoediting: integrating immunity’s roles in cancer suppression and promotion. Science.

[39] Shao XJ (2015). The down-regulation of microRNA-497 contributes to cell growth and cisplatin resistance through PI3K/Akt pathway in osteosarcoma. Cell. Physiol. Biochem.: Int. J. Exp. Cell. Physiol., Biochem., Pharmacol..

[40] Kudawara I (2013). Neoadjuvant and adjuvant chemotherapy with high-dose ifosfamide, doxorubicin, cisplatin and high-dose methotrexate in non-metastatic osteosarcoma of the extremities: a phase II trial in Japan. J. Chemother..

[41] Hassan M, Watari H, AbuAlmaaty A, Ohba Y, Sakuragi N (2014). Apoptosis and molecular targeting therapy in cancer. Biomed. Res. Int..

[42] Cotter TG (2009). Apoptosis and cancer: the genesis of a research field. Nat. Rev. Cancer.

[43] Kerr JF, Wyllie AH, Currie AR (1972). Apoptosis: a basic biological phenomenon with wide-ranging implications in tissue kinetics. Br. J. Cancer.

[44] Cryns V, Yuan J (1998). Proteases to die for. Genes Dev..

[45] Thornberry NA, Lazebnik Y (1998). Caspases: enemies within. Science.

[46] Antonsson B (1997). Inhibition of Bax channel-forming activity by Bcl-2. Science.

[47] Indran IR, Tufo G, Pervaiz S, Brenner C (2011). Recent advances in apoptosis, mitochondria and drug resistance in cancer cells. Biochim. Biophys. Acta.

[48] Teglund S, Toftgard R (2010). Hedgehog beyond medulloblastoma and basal cell carcinoma. Biochim. Biophys. Acta.

[49] Rovida E, Stecca B (2015). Mitogen-activated protein kinases and Hedgehog-GLI signaling in cancer: a crosstalk providing therapeutic opportunities?. Semin. Cancer Biol..

[50] Chi SM (2006). Activation of the hedgehog pathway in a subset of lung cancers. Cancer Lett..

[51] Nayak, A. et al. Nanoquinacrine induced apoptosis in cervical cancer stem cells through the inhibition of hedgehog-GLI1 cascade: role of GLI-1. *Sci. Rep.-UK* **6**, 20600 (2016). 10.1038/srep20600 PMC4742869 26846872

[52] Desch P (2010). Inhibition of GLI, but not Smoothened, induces apoptosis in chronic lymphocytic leukemia cells. Oncogene.

[53] Pan D (2012). Gli inhibitor GANT61 causes apoptosis in myeloid leukemia cells and acts in synergy with rapamycin. Leuk. Res..

[54] Chen XL (2008). Gli-1 siRNA induced apoptosis in Huh7 cells. World J. Gastroenterol..

[55] Katoh Y, Katoh M (2009). Hedgehog target genes: mechanisms of carcinogenesis induced by aberrant hedgehog signaling activation. Current molecular medicine.

[56] Bigelow RL (2004). Transcriptional regulation of bcl-2 mediated by the sonic hedgehog signaling pathway through gli-1. The Journal of biological chemistry.

[57] Wu JY (2011). Cyclopamine blocked the growth of colorectal cancer SW116 cells by modulating some target genes of Gli1 in vitro. Hepato-gastroenterology.

[58] Wu X (2017). Extra-mitochondrial prosurvival BCL-2 proteins regulate gene transcription by inhibiting the SUFU tumour suppressor. Nature cell biology.

[59] Gonnissen A, Isebaert S, Haustermans K (2015). Isebaert S, Haustermans K. Targeting the Hedgehog signaling pathway in cancer: beyond Smoothened. Oncotarget.

[60] Shahi MH, Holt R, Rebhun RB (2014). Blocking signaling at the level of GLI regulates downstream gene expression and inhibits proliferation of canine osteosarcoma cells. PLoS ONE.

[61] Yu HG (2008). Phosphoinositide 3-kinase/Akt pathway plays an important role in chemoresistance of gastric cancer cells against etoposide and doxorubicin induced cell death. Int. J. Cancer.

[62] Xu X, Wang B, Xu Y (2013). Expression of lysyl oxidase in human osteosarcoma and its clinical significance: a tumor suppressive role of LOX in human osteosarcoma cells. Int. J. Oncol..

[63] Tedesco I (2013). Dealcoholated red wine induces autophagic and apoptotic cell death in an osteosarcoma cell line. Food Chem. Toxicol.: Int. J. Publ. Br. Ind. Biol. Res. Assoc..

[64] Pandolfi S., Stecca B. Cooperative integration between HEDGEHOG-GLI signalling and other oncogenic pathways: implications for cancer therapy. *Exp. Rev. Mol. Med.* **17**, e5 (2015). 10.1017/erm.2015.3 PMC4836208 25660620

[65] Katoh Y, Katoh M (2009). Integrative genomic analyses on GLI1: positive regulation of GLI1 by Hedgehog-GLI, TGF beta-Smads, and RTK-PI3K-AKT signals, and negative regulation of GLI1 by Notch-CSL-HES/HEY, and GPCR-Gs-PKA signals. Int. J. Oncol..

[66] Sun Y (2015). Wogonoside protects against dextran sulfate sodium-induced experimental colitis in mice by inhibiting NF-kappa B and NLRP3 inflammasome activation. Biochem. Pharmacol..

