# Supplementary figures and images for: Glaucocalyxin A exerts anticancer effect on osteosarcoma by inhibiting GLI1 nuclear translocation via regulating PI3K/Akt pathway
Source: Cell Death Dis. 2018 Jun 13;9(6):708. doi: 10.1038/s41419-018-0684-9 (PMC5999605; doi:10.1038/s41419-018-0684-9)

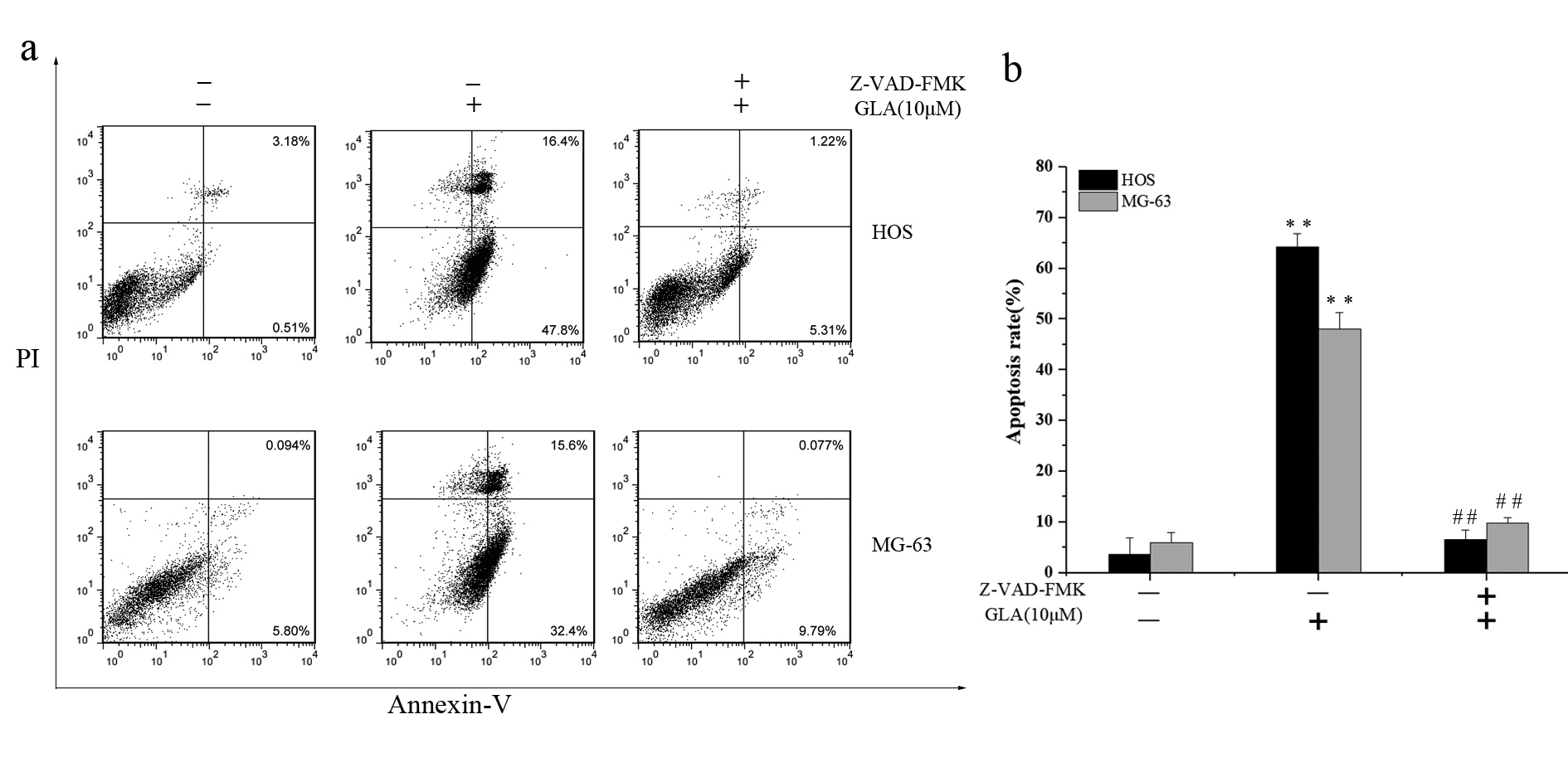

Supplement: Supplementary file 1 — Supplement Figure 1 [file 41419_2018_684_MOESM1_ESM.tif]

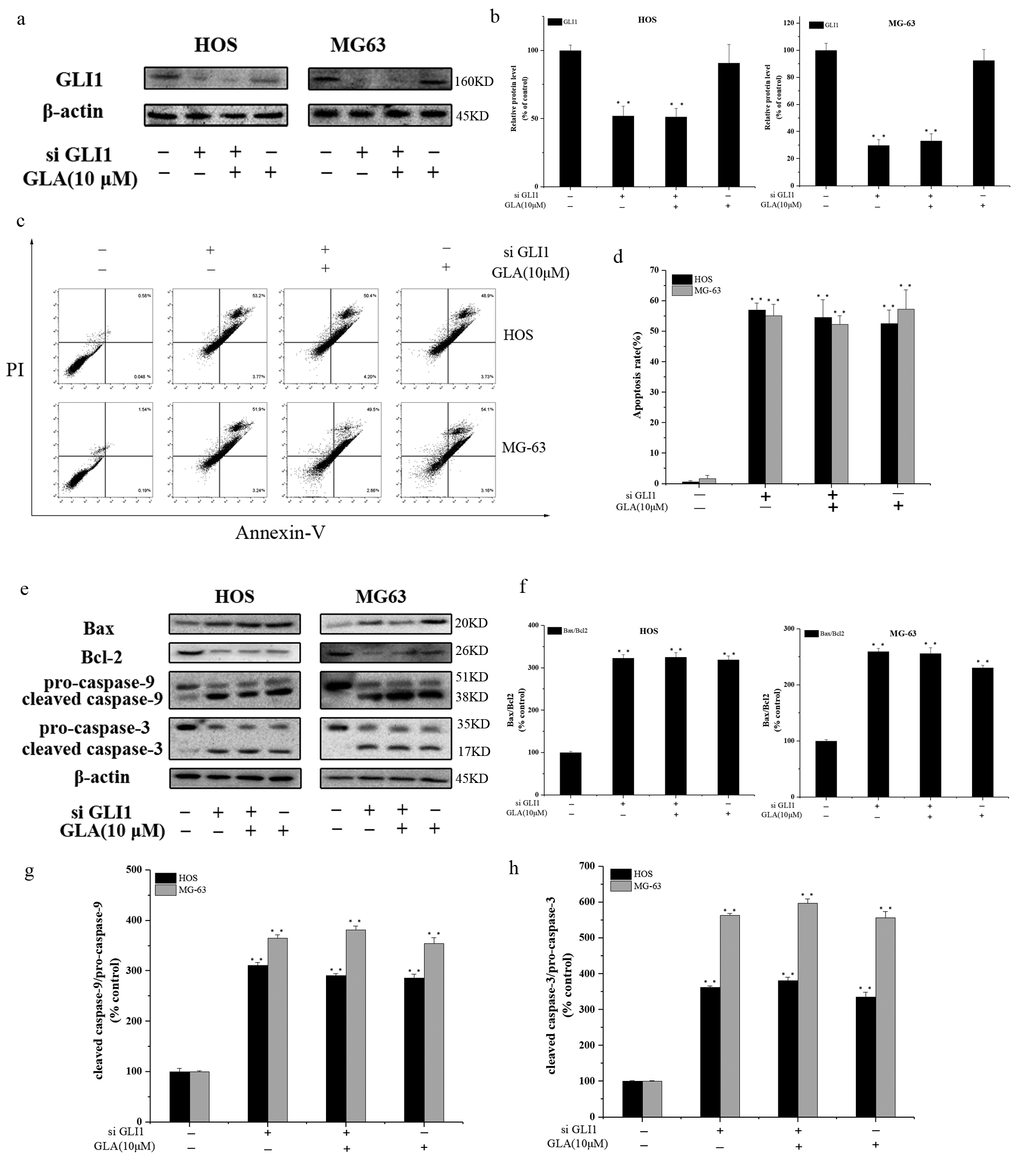

Supplement: Supplementary file 2 — Supplement Figure 2 [file 41419_2018_684_MOESM2_ESM.tif]

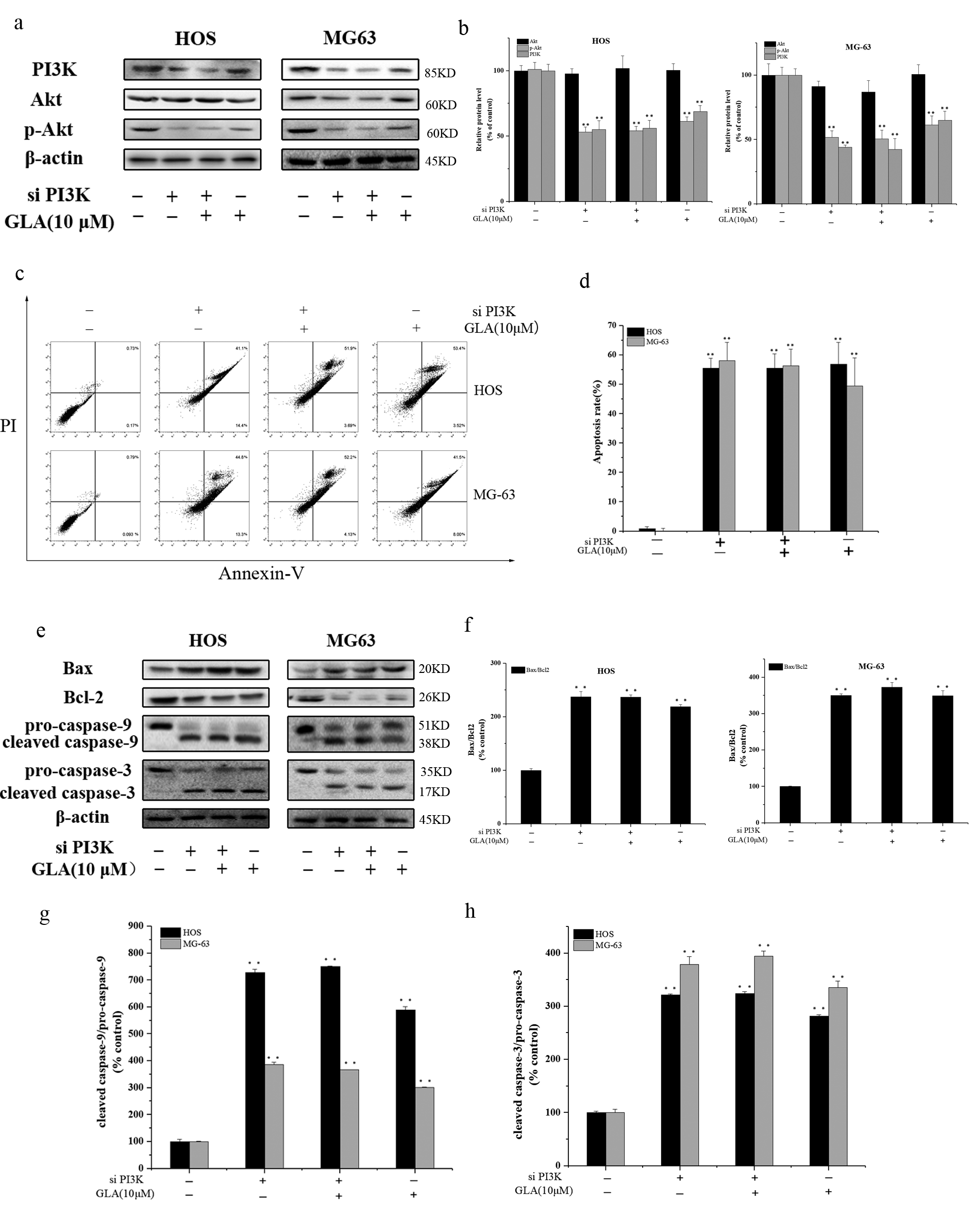

Supplement: Supplementary file 3 — Supplement Figure 3 [file 41419_2018_684_MOESM3_ESM.tif]
