# Supplementary material for: Glaucocalyxin A exerts anticancer effect on osteosarcoma by inhibiting GLI1 nuclear translocation via regulating PI3K/Akt pathway
Source: Cell Death Dis. 2018 Jun 13;9(6):708. doi: 10.1038/s41419-018-0684-9 (PMC5999605; doi:10.1038/s41419-018-0684-9)
Supplement: Supplementary file 4 — Supplementary figure legends [file 41419_2018_684_MOESM4_ESM.docx]

Supplement Figure 1 Glaucocalyxin A induced caspase-dependent apoptosis in human osteosarcoma cells. (a, b) After pre-treated caspase inhibitor Z-VAD-FMK 20 μM, the apoptotic rates of HOS and MG-63 cells induced by Glaucocalyxin A were detected by Annexin V/PI double-staining assay. The results are shown as means±SD from three independent experiments. ^**^P<0.01 compared with control group; ^##^P < 0.01 compared with Glaucocalyxin A (10 μM) group.

**Supplement** **Figure 2** Glaucocalyxin A induced apoptosis by inhibiting GLI1 activation in HOS and MG-63 cells. HOS and MG-63 cells were transfected with GLI1 siRNA for 8 h followed by with/without 10 µM Glaucocalyxin A for 24 h. (a) The protein expression of GLI1 in HOS and MG-63 cells was assessed by Western Blot. β-actin was used as an internal control. (b) Gray scale analysis was performed to determine the relative ratios of GLI1. The results are shown as means±SD from three independent experiments. **P<0.01 compared with control group. (c, d) The apoptotic rates of HOS and MG-63 cells induced by Glaucocalyxin A were detected by Annexin V/PI double-staining assay. (e) The protein expression of Bax, Bcl-2 pro-caspase-9, cleaved caspase-9, pro-caspase-3 and cleaved caspase-3 was assessed by Western Blot. β-actin was used as an internal control. (f-h) Gray scale analysis was performed to determine the relative ratios of Bax/Bcl-2, cleaved caspase-9/pro-caspase-9 and cleaved caspase-3/pro-caspase-3. The results are shown as means±SD from three independent experiments. ^**^P<0.01 compared with control group.

**Supplement Figure 3** Glaucocalyxin A induced apoptosis via inhibiting PI3K/Akt signaling pathway in HOS and MG-63 cells. HOS and MG-63 cells were transfected with PI3K siRNA for 8 h followed by with/without 10 µM Glaucocalyxin A for 24 h. (a) The protein expression of PI3K, Akt and p-Akt in HOS and MG-63 cells was assessed by Western Blot. β-actin was used as an internal control. (b) Gray scale analysis was performed to determine the relative ratios of PI3K, Akt and p-Akt. The results are shown as means±SD from three independent experiments. **P<0.01 compared with control group. (c, d) The apoptotic rates of HOS and MG-63 cells induced by Glaucocalyxin A were detected by Annexin V/PI double-staining assay. (e) The protein expression of Bax, Bcl-2 pro-caspase-9, cleaved caspase-9, pro-caspase-3 and cleaved caspase-3 was assessed by Western Blot. β-actin was used as an internal control. (f-h) Gray scale analysis was performed to determine the relative ratios of Bax/Bcl-2, cleaved caspase-9/pro-caspase-9 and cleaved caspase-3/pro-caspase-3. The results are shown as means±SD from three independent experiments. ^**^P<0.01 compared with control group.
